# Draft Genome Sequences of Two Highly Erythromycin-Resistant *Streptococcus gallolyticus* subsp. *gallolyticus* Isolates Containing a Novel Tn*916*-Like Element, Tn*6331*

**DOI:** 10.1128/genomeA.00226-17

**Published:** 2017-04-20

**Authors:** Stanimir Kambarev, Frédéric Pecorari, Stéphane Corvec

**Affiliations:** a CRCINA, Inserm, CNRS, Université d’Angers, Université de Nantes, Nantes, France; b CRCINA, Inserm, Université d’Angers, Université de Nantes, Nantes, France; c Service de Bactériologie & Hygiène Hospitalière, CHU de Nantes, Nantes, France

## Abstract

Recently, we reported the draft genome sequence of *Streptococcus gallolyticus* NTS31106099. It was found to contain a previously unknown putative Tn*916*-like conjugative transposon, Tn*6263*. Here, we report the draft genome sequences of two other clinical isolates, NTS31301958 and NTS31307655. Both of them contain another novel element, Tn*6331*, which is highly similar to Tn*6263*.

## GENOME ANNOUNCEMENT

*Streptococcus gallolyticus* subsp. *gallolyticus* is a Gram-positive gastrointestinal commensal and opportunistic pathogen often found in animals and humans. The clinical relevance of *S. gallolyticus* subsp. *gallolyticus* (previously *Streptococcus bovis* biotype I) to humans is due to its ability to occasionally cause various clinical entities, mainly infective endocarditis and bacteremia (1, 2). Furthermore, the bacterium has been shown to be closely associated with the presence of colorectal malignancy (3). However, the virulence arsenal and pathogenesis of *S. gallolyticus* subsp. *gallolyticus* remain poorly understood (2, 4, 5). Its first sequenced genomes were released almost a decade ago, and early comparative analysis has suggested an active involvement of *S. gallolyticus* subsp. *gallolyticus* in horizontal gene transfer with other rumen or gut *Firmicutes*, such as enterococci, lactobacilli, bacilli, and clostridia (6–9). Surprisingly, even tough epidemiological studies have shown a high prevalence of erythromycin-resistant isolates (10, 11) and the number of sequenced *S. gallolyticus* subsp. *gallolyticus* genomes continues to grow steadily, mobile elements encoding macrolide resistance determinants have not yet been described in this organism. Only a limited number of such elements have been characterized in the other *S. gallolyticus* subspecies, *S. gallolyticus* subsp. *pasteurianus* (12–15). Recently, we reported the draft genome sequence of highly erythromycin-resistant clinical isolate *S. gallolyticus* subsp. *gallolyticus* NTS31106099 (16). It was found to contain previously unknown putative Tn*916*-like conjugative transposon, Tn*6263*, which harbors an aminoglycoside/macrolide resistance cluster [*aph(3′)-III*→*ant(6)-Ia*→*ermB*]. Here, we present the draft genome sequences of two other highly erythromycin-resistant isolates of *S. gallolyticus* subsp. *gallolyticus*, NTS31301958 and NTS31307655. They contain another previously unknown element, Tn*6331*, which is highly similar to Tn*6263* and harbors an identical resistance cluster.

Cultures and genomic DNA were prepared as described elsewhere (16). Genome fragmentation was performed with Bioruptor Standard (Diagenode). About 1 µg of fragmented DNA (200 to 300 bp) was used for preparation of sequencing libraries using the NEBNext Ultra DNA library prep kit for Illumina (NEB) and sequenced on a MiSeq sequencer (Illumina). The draft sequences of NTS31301958 and NTS31307655 were assembled *de novo* from 1,499,382 and 1,710,880 high-quality 150-bp paired-end reads, respectively, using Velvet 1.2.10 (17) and VelvetOptimiser 2.2.5 (18). The resulting sets of contigs were reordered against the complete genome of strain UCN34 (7) using Mauve 1.2.10 (19) and annotated through the NCBI Prokaryotic Genome Automatic Annotation Pipeline (20).

*De novo* assembly of the NTS31301958 genome resulted in a set of 22 contigs, with an average coverage of 96× and an *N*_50_ of 1,180 kb. The draft sequence has a total length of 2,330,998 bp and G+C content of 37.5%. About 2,253 coding sequences (CDSs) were automatically annotated, including 42 pseudogenes and 67 RNA genes. The draft genome of isolate NTS31307655 consists of 30 contigs, with an average coverage of 103× and an *N*_50_ of 272 kb. The sequence is 2,332,206 bp long and has a G+C content of 37.5%. Annotation revealed 2,254 CDSs, 49 pseudogenes, and 67 RNA genes.

### Accession number(s).

The draft genomes of *S. gallolyticus* subsp. *gallolyticus* NTS31301958 and NTS31307655 sequenced under this project have been deposited at DDBJ/EMBL/GenBank under the accession numbers MAMV00000000 and LXFC00000000, respectively. The versions described in this paper are MAMV01000000 and LXFC01000000.
